# Supplementary material for: The Role of Reduced Methionine in Mediating the Metabolic Responses to Protein Restriction Using Different Sources of Protein
Source: Nutrients. 2021 Jul 29;13(8):2609. doi: 10.3390/nu13082609 (PMC8399679; doi:10.3390/nu13082609)
Supplement: Supplementary file 1 [file nutrients-13-02609-s001.zip › nutrients-1314433-supplementary.pdf]

Table S1 – Essential amino acid (EAA) and non-essential amino acid (NEAA) composition of the casein and soy proteins used to formulate the experimental diets

		Casein, gm%	Soy, gm%
EAA	His[1]	2.6	2.6
	Ile	4.3	4.9
	Leu	9.0	8.2
	Lys	7.5	6.3
	Met	2.9	1.3
	Phe	4.8	5.2
	Thr	4.1	3.7
	Trp	1.2	1.4
	Val	5.3	5
NEAA	Ala	2.9	4.3
	Arg	3.4	7.6
	Asn	4	NA
	Asp	2.9	11.6
	Cys	0.7	1.3
	Gln	9.8	NA
	Glu	11.9	19.1
	Gly	1.7	4.2
	Pro	10.1	5.1
	Ser	5.7	5.2
	Tyr	5.2	3.8
Total %		100	100.8

[1]Three letter abbreviations of amino acids. Sulfur amino acids, methionine and cysteine highlighted in yellow.

Table S2 – Primers used for real time PCR analysis.

Gene	Forward	Reverse
Scd1	GTGCCGTGGGCGAGGGCTTC	AGCCCAAAGCTCAGCTACTCTT
Fasn	TCCTGGAACGAGAACACGATCT	GAGACGTGTCACTCCTGGACTTG
Fgf21	TGACACCCAGGATTTGAATGAC	GCAGCCAATGATGTGTGCTTAC
Asns	GGGGGCCTGGACTCGAGCTT	TTGCCACCTTTCTAGCGGCCA
Psat1	CAGTGGAGCGCCAGAATAGAA	CCTGTGCCCCTTCAAGGAG
Mgst3	ATGGCTGTCCTCTCTAAGGAG	CTCTACCTTGTACTTCTTGCGG
Ucp1	GATCCAAGGTGAAGGCCAGG	GTTGACAAGCTTTCTGTGGTGG
Cox7a1	CAGCGTCATGGTCAGTCTGT	AGAAAACCGTGTGGCAGAGA
Cox8b	AGTTCACAGTGGTTCCCAAAG	ACCATGAAGCCAACGACTATG
Cidea	ATCACAACTGGCCTGGTTACG	TACTACCCGGTGTCCATTTCT
Cyclophilin	CTTCGAGCTGTTTGCAGACAAAGT	AGATGCCAGGACCTGTATGCT

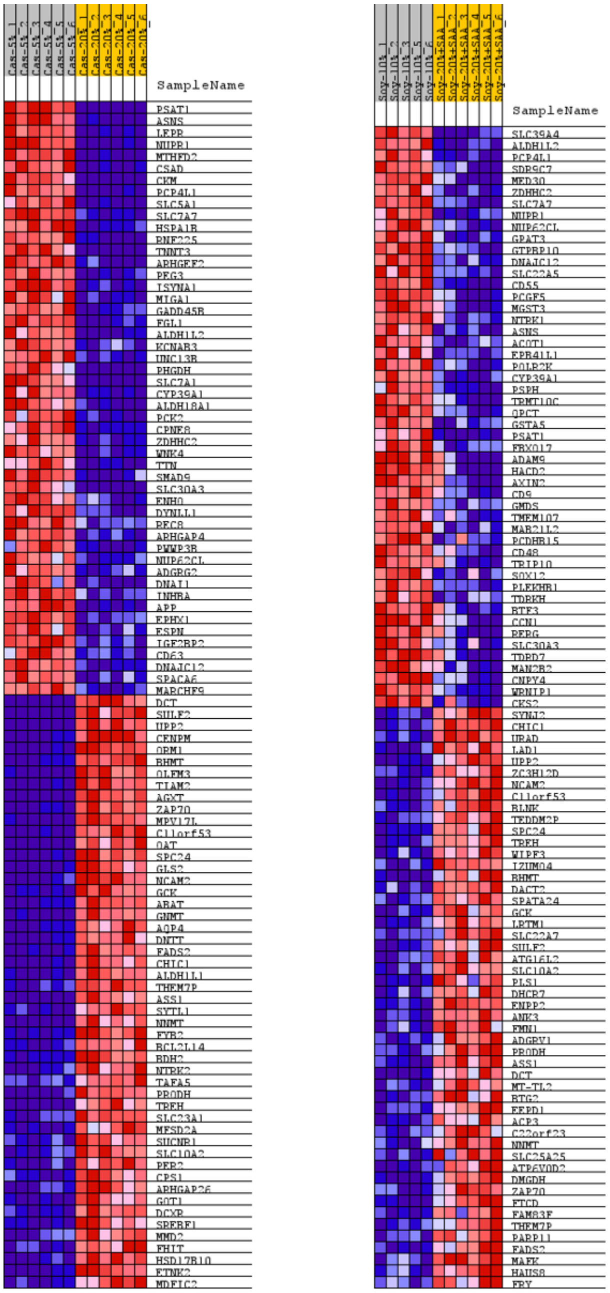

1A **1B**

Figure S1: Gene Set Enrichment Analysis (GSEA) of hepatic gene expression in mice fed sulfur amino acid-matched control diets formulated from casein (Cas-20%) or soy (Soy-20%+SAA), and sulfur amino acid-matched protein restricted diets formulated from casein (Cas-5%) or soy (Soy-10%). The heat maps are shown for the top 50 up-regulated and down-regulated genes for the Cas-5% to Cas-20% (**1A**) and Soy-10% to Soy-20%+SAA comparisons (**1B**). The heat maps present the normalized enrichment scores for each mouse and the corresponding individual genes within the gene set [blue = downregulation, red = upregulation, (FDR > 0.1)].

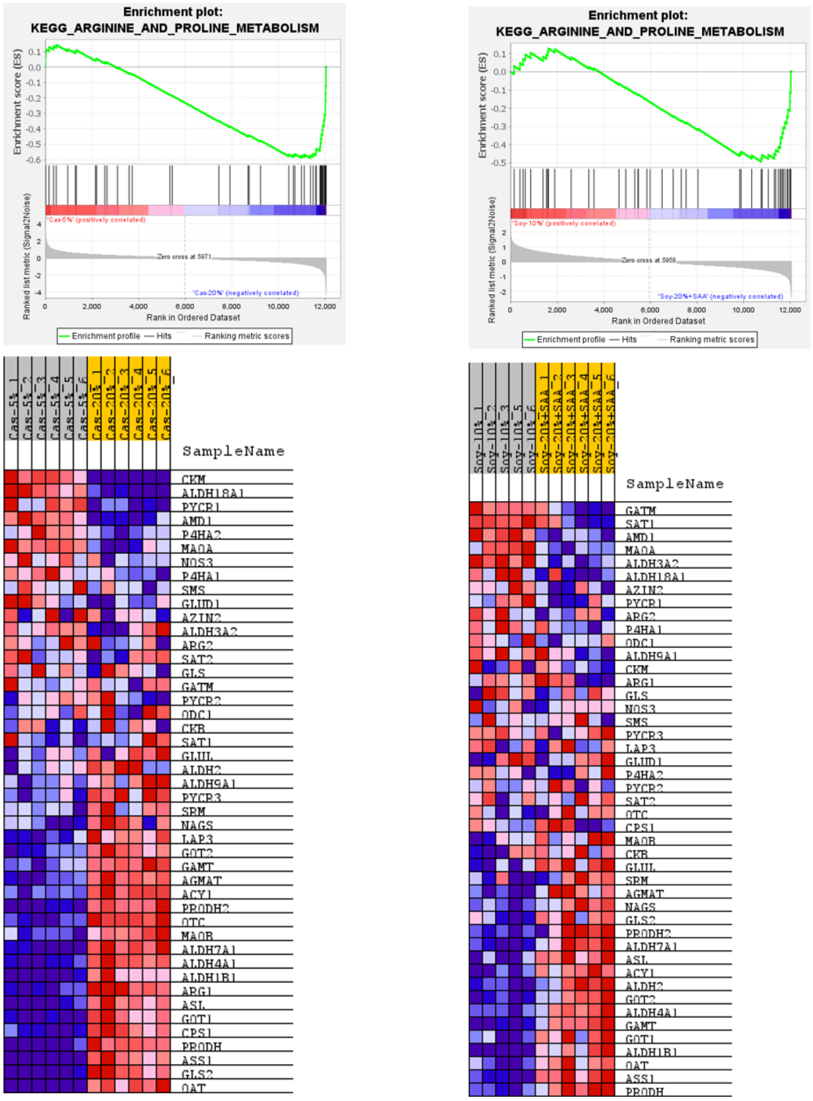

2A **2B**

Figure S2: Gene set enrichment analysis (GSEA) of hepatic gene expression in mice fed sulfur amino acid-matched control diets formulated from casein (Cas-20%) or soy (Soy-20%+SAA), and sulfur amino acid-matched protein restricted diets formulated from casein (Cas-5%) or soy (Soy-10%). The enrichment plots and corresponding heat maps are shown for the top-scoring KEGG Arginine-Proline Metabolism gene set for the Cas-5% to Cas-20% (**2A**) and Soy-10% to Soy-20%+SAA comparisons (**2B**) . Enrichment was computed as described in the Material and Methods and the individual contributions of pathway genes to the pathway enrichment signal were visualized via enrichment plots depicting the trajectory of a normalized pathway enrichment score against the rank of the pathway genes in the context of the full gene list. Accompanying heat maps present the normalized enrichment scores for individual genes within the gene set [blue = downregulation, red = upregulation, gray = not significant (FDR > 0.1)].

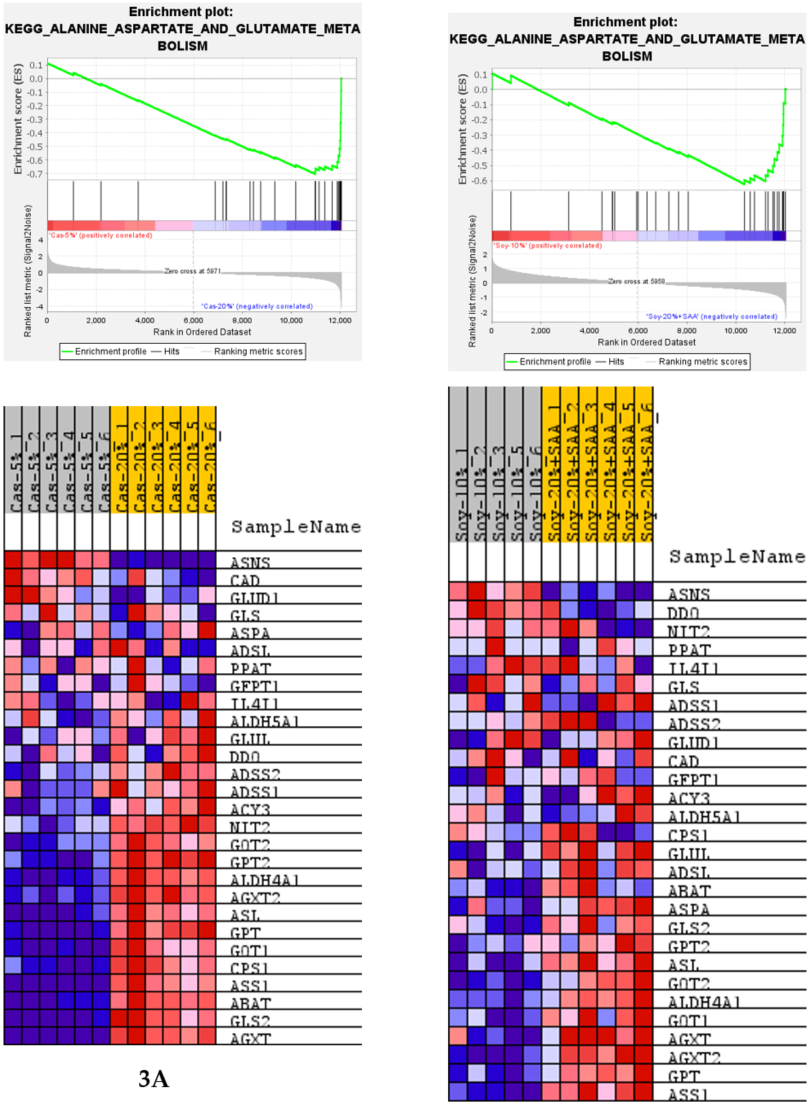

3A **3B**

Figure S3: Gene set enrichment analysis (GSEA) of hepatic gene expression in mice fed sulfur amino acid-matched control diets formulated from casein (Cas-20%) or soy (Soy-20%+SAA), and sulfur amino acid-matched protein restricted diets formulated from casein (Cas-5%) or soy (Soy-10%). The enrichment plots and corresponding heat maps are shown for the top-scoring KEGG Alanine-Aspartate-Glutamate Metabolism gene set for the Cas-5% to Cas-20% (**3A**) and Soy-10% to Soy-20%+SAA comparisons (**3B**) . Enrichment was computed as described in the Material and Methods and the individual contributions of pathway genes to the pathway enrichment signal were visualized via enrichment plots depicting the trajectory of a normalized pathway enrichment score against the rank of the pathway genes in the context of the full gene list. Accompanying heat maps present the normalized enrichment scores for individual genes within the gene set [blue = downregulation, red = upregulation, gray = not significant (FDR > 0.1)].

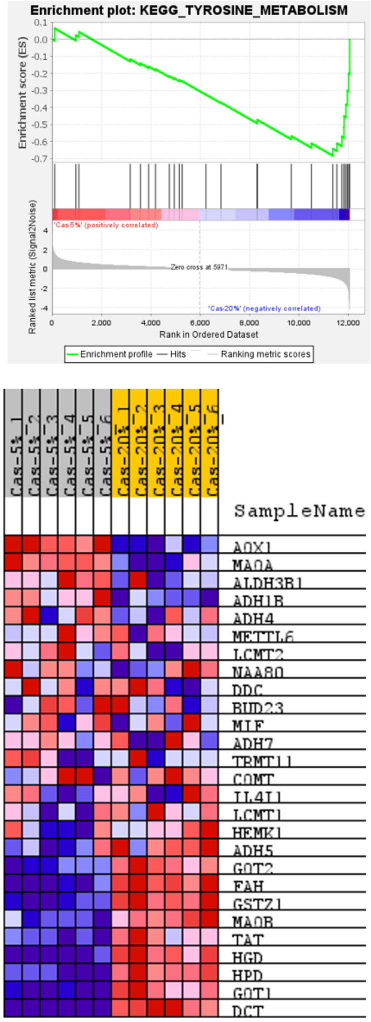

4A

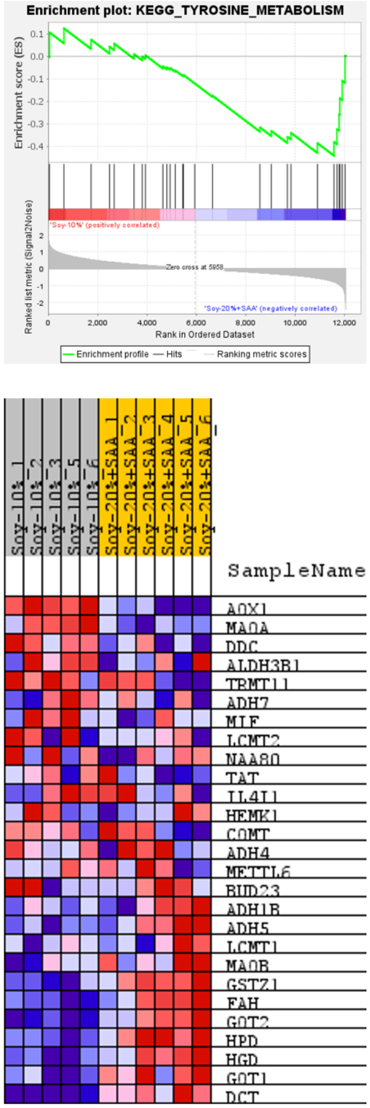

4B

Figure S4: Gene set enrichment analysis (GSEA) of hepatic gene expression in mice fed sulfur amino acid-matched control diets formulated from casein (Cas-20%) or soy (Soy-20%+SAA), and sulfur amino acid-matched protein restricted diets formulated from casein (Cas-5%) or soy (Soy-10%). The enrichment plots and corresponding heat maps are shown for the top-scoring KEGG Tyrosine Metabolism gene set for the Cas-5% to Cas-20% (**4A**) and Soy-10% to Soy-20%+SAA comparisons (**4B**) . Enrichment was computed as described in the Material and Methods and the individual contributions of pathway genes to the pathway enrichment signal were visualized via enrichment plots depicting the trajectory of a normalized pathway enrichment score against the rank of the pathway genes in the context of the full gene list. Accompanying heat maps present the normalized enrichment scores for individual genes within the gene set [blue = downregulation, red = upregulation, gray = not significant (FDR > 0.1)].

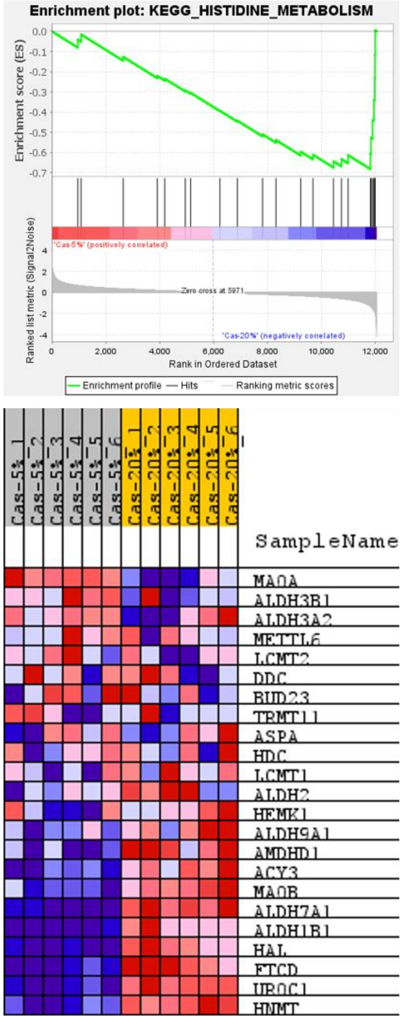

5A

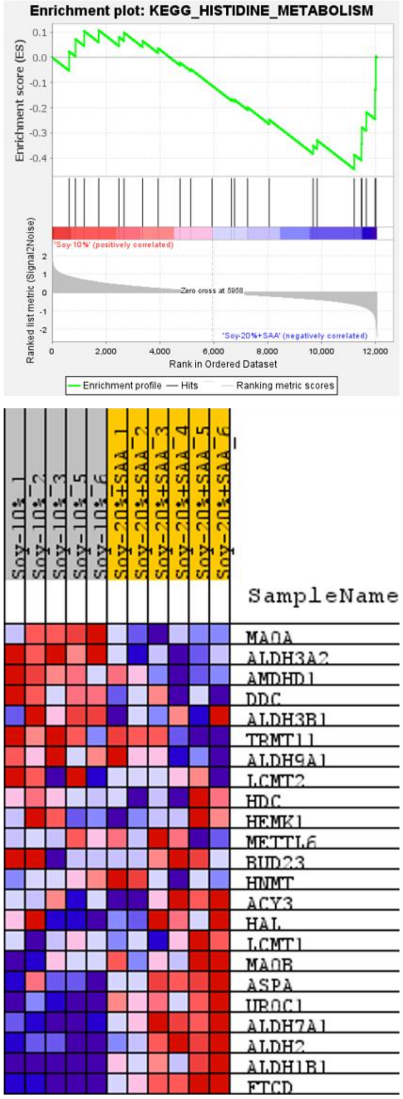

5B

Figure S5: Gene set enrichment analysis (GSEA) of hepatic gene expression in mice fed sulfur amino acid-matched control diets formulated from casein (Cas-20%) or soy (Soy-20%+SAA), and sulfur amino acid-matched protein restricted diets formulated from casein (Cas-5%) or soy (Soy-10%). The enrichment plots and corresponding heat maps are shown for the top-scoring KEGG Histidine Metabolism gene set for the Cas-5% to Cas-20% (**5A**) and Soy-10% to Soy-20%+SAA comparisons (**5B**) . Enrichment was computed as described in the Material and Methods and the individual contributions of pathway genes to the pathway enrichment signal were visualized via enrichment plots depicting the trajectory of a normalized pathway enrichment score against the rank of the pathway genes in the context of the full gene list. Accompanying heat maps present the normalized enrichment scores for individual genes within the gene set [blue = downregulation, red = upregulation, gray = not significant (FDR > 0.1)].

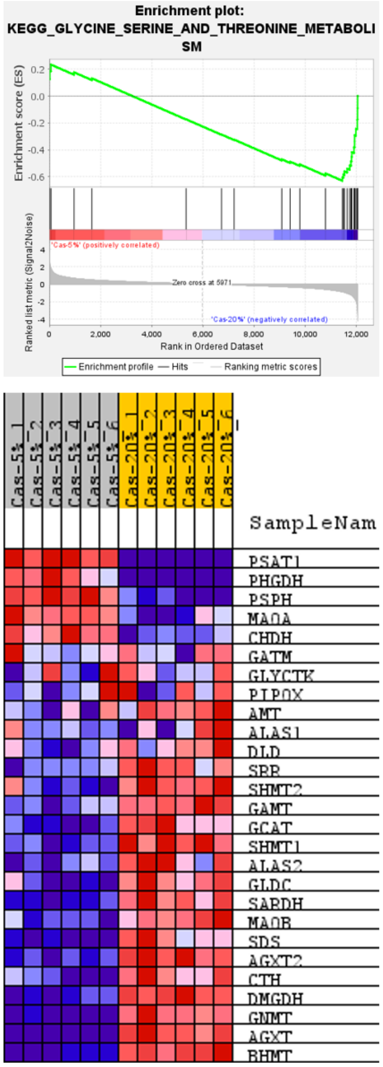

6A

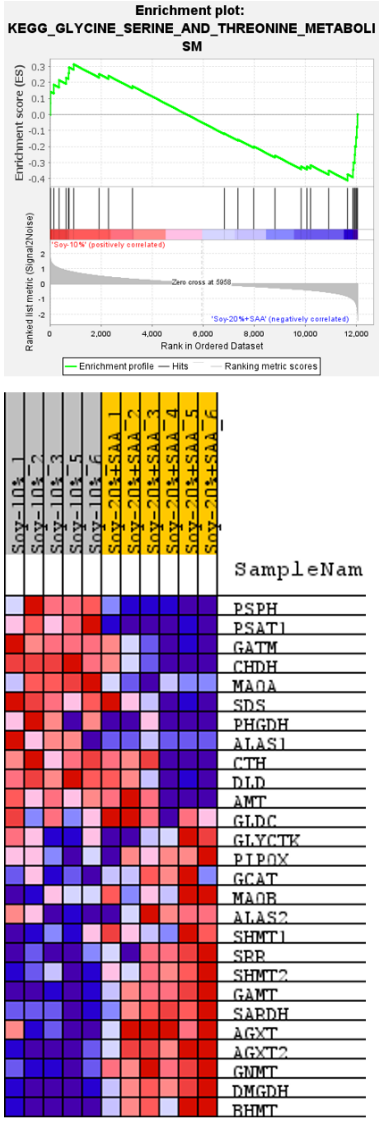

6B

Figure S6: Gene set enrichment analysis (GSEA) of hepatic gene expression in mice fed sulfur amino acid-matched control diets formulated from casein (Cas-20%) or soy (Soy-20%+SAA), and sulfur amino acid-matched protein restricted diets formulated from casein (Cas-5%) or soy (Soy-10%). The enrichment plots and corresponding heat maps are shown for the top-scoring KEGG Glycine-Serine-Threonine Metabolism gene set for the Cas-5% to Cas-20% (**6A**) and Soy-10% to Soy-20%+SAA comparisons (**6B**) . Enrichment was computed as described in the Material and Methods and the individual contributions of pathway genes to the pathway enrichment signal were visualized via enrichment plots depicting the trajectory of a normalized pathway enrichment score against the rank of the pathway genes in the context of the full gene list. Accompanying heat maps present the normalized enrichment scores for individual genes within the gene set [blue = downregulation, red = upregulation, gray = not significant (FDR > 0.1)].

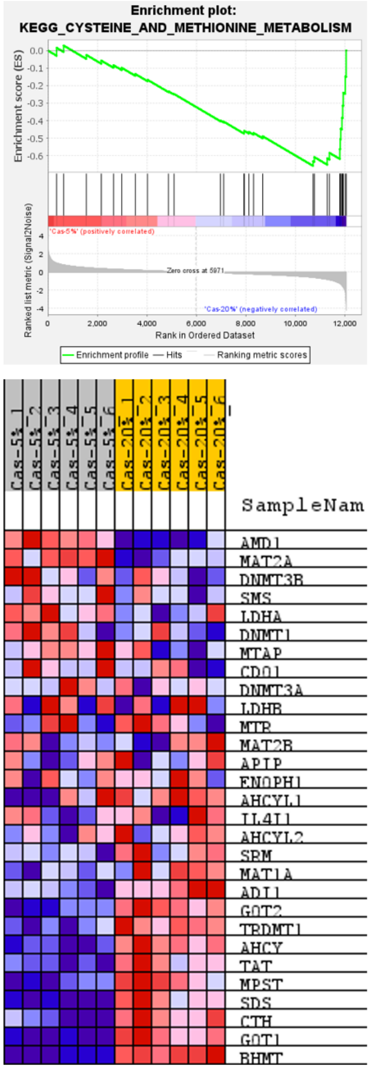

7A

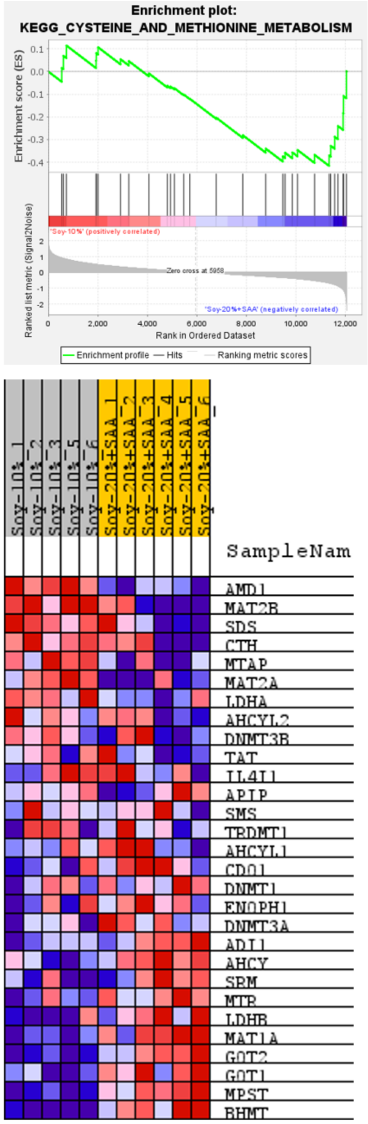

7B

Figure S7: Gene set enrichment analysis (GSEA) of hepatic gene expression in mice fed sulfur amino acid-matched control diets formulated from casein (Cas-20%) or soy (Soy-20%+SAA), and sulfur amino acid-matched protein restricted diets formulated from casein (Cas-5%) or soy (Soy-10%). The enrichment plots and corresponding heat maps are shown for the top-scoring KEGG Cysteine-Methionine Metabolism gene set for the Cas-5% to Cas-20% (**7A**) and Soy-10% to Soy-20%+SAA comparisons (**7B**) . Enrichment was computed as described in the Material and Methods and the individual contributions of pathway genes to the pathway enrichment signal were visualized via enrichment plots depicting the trajectory of a normalized pathway enrichment score against the rank of the pathway genes in the context of the full gene list. Accompanying heat maps present the normalized enrichment scores for individual genes within the gene set [blue = downregulation, red = upregulation, gray = not significant (FDR > 0.1)].

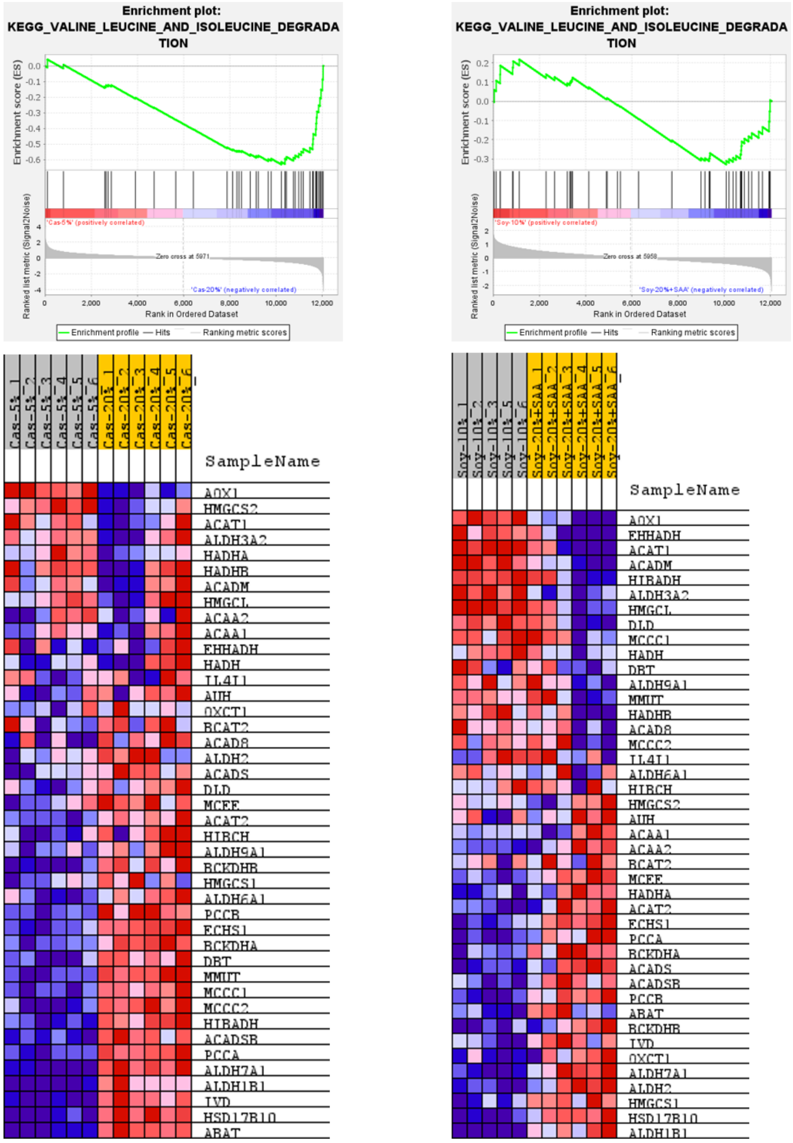

8A **8B**

Figure S8: Gene set enrichment analysis (GSEA) of hepatic gene expression in mice fed sulfur amino acid-matched control diets formulated from casein (Cas-20%) or soy (Soy-20%+SAA), and sulfur amino acid-matched protein restricted diets formulated from casein (Cas-5%) or soy (Soy-10%). The enrichment plots and corresponding heat maps are shown for the top-scoring KEGG Valine-Isoleucine-Leucine Degradation gene set for the Cas-5% to Cas-20% (**8A**) and Soy-10% to Soy-20%+SAA comparisons (**8B**) . Enrichment was computed as described in the Material and Methods and the individual contributions of pathway genes to the pathway enrichment signal were visualized via enrichment plots depicting the trajectory of a normalized pathway enrichment score against the rank of the pathway genes in the context of the full gene list. Accompanying heat maps present the normalized enrichment scores for individual genes within the gene set [blue = downregulation, red = upregulation, gray = not significant (FDR > 0.1)].

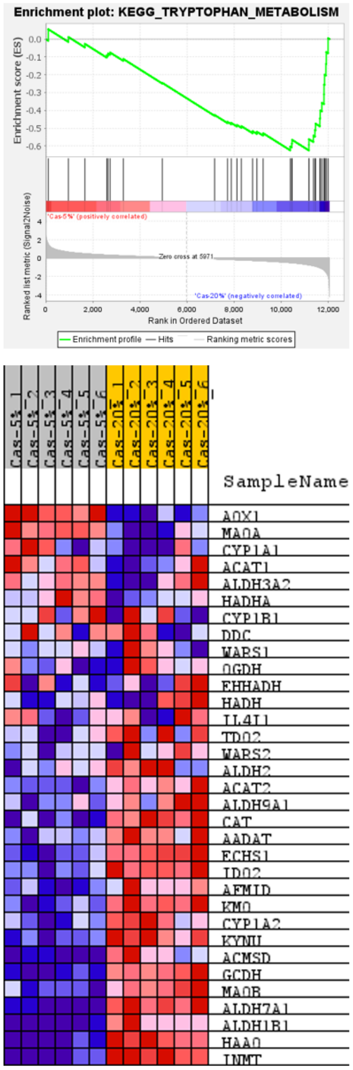

9A

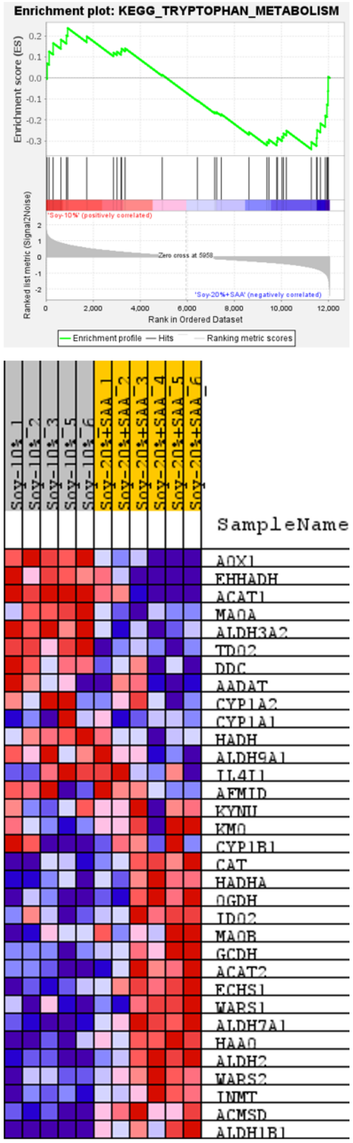

9B

Figure S9: Gene set enrichment analysis (GSEA) of hepatic gene expression in mice fed sulfur amino acid-matched control diets formulated from casein (Cas-20%) or soy (Soy-20%+SAA), and sulfur amino acid-matched protein restricted diets formulated from casein (Cas-5%) or soy (Soy-10%). The enrichment plots and corresponding heat maps are shown for the top-scoring KEGG Tryptophan Metabolism gene set for the Cas-5% to Cas-20% (**9A**) and Soy-10% to Soy-20%+SAA comparisons (**9B**) . Enrichment was computed as described in the Material and Methods and the individual contributions of pathway genes to the pathway enrichment signal were visualized via enrichment plots depicting the trajectory of a normalized pathway enrichment score against the rank of the pathway genes in the context of the full gene list. Accompanying heat maps present the normalized enrichment scores for individual genes within the gene set [blue = downregulation, red = upregulation, gray = not significant (FDR > 0.1)].
